# Sulf1 has ligand-dependent effects on canonical and non-canonical Wnt signalling

**DOI:** 10.1242/jcs.164467

**Published:** 2015-04-01

**Authors:** Simon W. Fellgett, Richard J. Maguire, Mary Elizabeth Pownall

**Affiliations:** Biology Department, University of York, York YO10 5YW, UK

**Keywords:** Morphogen, Development, Cell signalling, Pronephros, 6-O-endosulfatase, Heparan sulfate, HSPG, Wnt

## Abstract

Wnt signalling plays essential roles during embryonic development and is known to be mis-regulated in human disease. There are many molecular mechanisms that ensure tight regulation of Wnt activity. One such regulator is the heparan-sulfate-specific 6-O-endosulfatase Sulf1. Sulf1 acts extracellularly to modify the structure of heparan sulfate chains to affect the bio-availability of Wnt ligands. Sulf1 could, therefore, influence the formation of Wnt signalling complexes to modulate the activation of both canonical and non-canonical pathways. In this study, we use well-established assays in *Xenopus* to investigate the ability of Sulf1 to modify canonical and non-canonical Wnt signalling. In addition, we model the ability of Sulf1 to influence morphogen gradients using fluorescently tagged Wnt ligands in ectodermal explants. We show that Sulf1 overexpression has ligand-specific effects on Wnt signalling: it affects membrane accumulation and extracellular levels of tagged Wnt8a and Wnt11b ligands differently, and inhibits the activity of canonical Wnt8a but enhances the activity of non-canonical Wnt11b.

## INTRODUCTION

Heparan sulfate proteoglycans (HSPGs) are present in all animals and play key roles in cell–cell signalling pathways, including an essential role in Wnt signalling (Baeg et al., 2001; Lin and Perrimon, 1999). The structure of heparan sulfate chains can be modified post-synthetically by the extracellular heparan sulfate 6-O endosulfatases, Sulf1 and Sulf2, which remove a sulfate group from N-sulfated glucosamine in highly sulfated regions of heparan sulfate glycosaminoglycan (GAG) chains (Esko and Selleck, 2002; Häcker et al., 2005). Sulf activity has been shown to reduce heparin binding to Wnt8a, and the prevailing model is that Sulf1 promotes Wnt signalling by releasing Wnt ligands from cell surface HSPGs, thus making them more available for presentation to the Frizzled receptor (Ai et al., 2003). The ability of Sulf enzymes to enhance canonical Wnt signalling is supported by several other reports (Dhoot et al., 2001; Hitchins et al., 2013; Nawroth et al., 2007; Tang and Rosen, 2009; Tran et al., 2012); however, not all data are consistent with Sulf1 enhancing Wnt signalling. For instance, *Drosophila* mutants deficient in Sulf have a wing phenotype consistent with elevated Wg (a *Drosophila* Wnt) (Kleinschmit et al., 2010; You et al., 2011), pointing to a role for Sulf in restricting Wnt signalling in this context.

A thorough review of Wnt signalling has been published recently (Hoppler and Moon, 2014). Wnt proteins have been classified as either being canonical or non-canonical ligands (Du et al., 1995), but this distinction is questionable because both Wnt5a and Wnt11b (the classic non-canonical Wnts) can activate canonical Wnt signalling (Mikels and Nusse, 2006; Tao et al., 2005) in the presence of the necessary receptors and pathway specific co-receptors (Yamamoto et al., 2008a). For instance, Ror2 is a co-receptor that promotes non-canonical signalling (Schambony and Wedlich, 2007), whereas LRP6 is essential for canonical Wnt signalling (Cadigan and Liu, 2006; Tamai et al., 2000). Regulation of Wnt signalling can occur at the level of these receptor complexes; for instance, Cthrc1 is a pro-migratory protein that associates with the Wnt–Frz–Ror2 receptor complex to promote activation of the non-canonical Wnt pathway (Yamamoto et al., 2008b). In addition, Dkk1 interacts with the Wnt–Frz–LRP6 complex to inhibit canonical Wnt signalling (MacDonald et al., 2004). Sulf enzymes might also regulate Wnt signalling at this level because HSPGs are known to be important for receptor–ligand interactions (Turnbull et al., 2001), and Sulf1 has been shown to affect the association of signalling complexes (Ai et al., 2003; Freeman et al., 2008; Wang et al., 2004).

In this study, we present evidence that Sulf1 can differentially regulate Wnt signalling in a ligand-dependent manner. Using an axis-inducing assay in *Xenopus* embryos (Du et al., 1995), we find that Sulf1 inhibits canonical Wnt signalling by modulating the potent activator of this pathway, Wnt8a. However, we also show that Sulf1 does not affect axis induction by Wnt3a, indicating that Sulf1 does not inhibit all canonical Wnt signalling in this context. In addition, we demonstrate that Sulf1 enhances Wnt11b activity in non-canonical assays for convergent extension and subcellular localisation of Dishevelled2 (Dvl2, hereafter denoted Dvl) (Miller et al., 1999). We conclude that the effects of Sulf1 are ligand specific and reflect the diverse molecular mechanisms regulated by HSPGs, such as the establishment of signalling complexes and the formation of morphogen gradients.

## RESULTS

### Sulf1 inhibits Wnt8a induction of a secondary axis in *Xenopus*

**Fig. 1.**
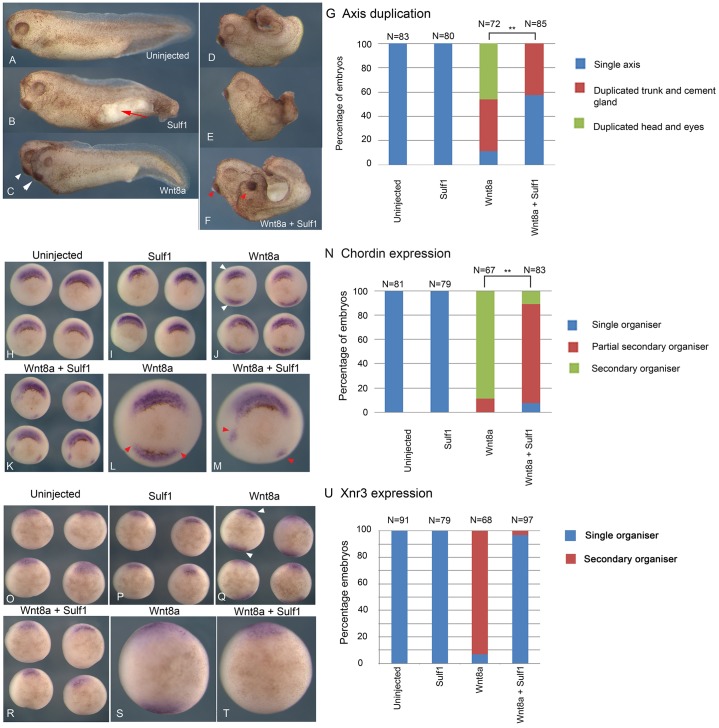
**Sulf1 inhibits the ability of Wnt8a to activate canonical Wnt signalling.** (A–M) *Xenopus laevis* embryos were microinjected with mRNA encoding *Wnt8a* (5 pg) and/or *Sulf1* (1 ng) into a single ventral blastomere at the four-cell stage. (A–F) Whole embryo phenotypes of (A) uninjected control embryo, and embryos injected with (B) *Sulf1*, (C) *Wnt8a* or (D–F) *Sulf1* and *Wnt8a*. White arrowheads in C mark a fully duplicated axis; red arrowheads in F mark duplicated trunk and cement gland. (G) The data shown in A–F is quantified in G. **P<0.01 (Chi squared test). (H–M) *In situ* hybridisation shows the expression of *chordin* at NF stage 10.5. (H) Uninjected control embryos; (I–K) embryos injected with (I) *Sulf1*, (J) *Wnt8a* and (K) *Sulf1* and *Wnt8a*. (L) Wnt8a induces an ectopic domain of *chordin* expression. (M) Sulf1 inhibits the ability of Wnt8a to induce ectopic *chordin* resulting in the formation of a partial secondary organiser domain. The red arrowheads in L and M mark the edges of the ectopic *chordin* domains. (N) The data shown in H–M is quantified in N, **P<0.01 (Chi squared test). (O–U) *In situ* hybridisation shows the expression of *Xnr3* at NF stage 10. (O) Uninjected control embryos; (P–T) embryos injected with (P) *Sulf1*, (Q) *Wnt8a* and (R) *Sulf1* and *Wnt8a*. Enlarged images of embryos shown in Q and R are shown in S and T. The data shown in O–T is quantified in (U). *N*, number of embryos.

In order to investigate the activity of Sulf1 in modulating canonical Wnt signalling in *Xenopus*, mRNAs encoding Wnt8a and Sulf1 were microinjected into a single ventral blastomere at the four-cell stage and examined for phenotype at Nieuwkoop–Faber (NF) stage 36 (Nieuwkoop and Faber, 1967). Overexpression of Sulf1 alone disrupted gastrulation, resulting in an open blastopore, but had no effect on axis duplication (Fig. 1B, red arrow marks exposed yolk). Overexpression of Wnt8a in a single ventral blastomere results in the formation of a secondary axis in 90% of embryos, half of which have duplicated heads (Fig. 1C, white arrowheads). Overexpression of Sulf1 together with Wnt8a inhibited secondary axis formation and led to severe truncation of the embryo (Fig. 1D–F); although duplicated cement glands were present in some embryos there were no duplicated heads in the presence of Sulf1 (Fig. 1F, see red arrowheads). The results shown in Fig. 1A–F are quantified in Fig. 1G. These data show that in contrast to the accepted model, in this assay, Sulf1 inhibits rather than enhances canonical Wnt signalling (Ai et al., 2003; Tang and Rosen, 2009; Tran et al., 2012).

Secondary axis formation mediated by Wnt8a results from a duplication of Spemann's organiser, a region of the embryo that expresses bone morphogenetic protein (BMP) inhibitors such as *chordin* in response to dorsal signals including canonical Wnt signalling. To examine the effects of Sulf1 on organiser duplication, the expression of *chordin* was analysed at mid gastrula stage. Overexpression of Sulf1 alone in a single ventral blastomere had no effect on *chordin* (Fig. 1I), whereas overexpression of Wnt8a induced ectopic *chordin* expression in 90% of injected embryos (Fig. 1J, see white arrowheads). Overexpression of Sulf1 together with Wnt8a inhibited the ability of Wnt8a to induce an ectopic domain of *chordin* expression (Fig. 1K), however, small patches of peripheral *chordin* expression were still detected in some embryos (Fig. 1M) and classified as a ‘partial organiser’ for quantification (see Fig. 1N). We also analysed the expression of *Xnr3* (also known as *nodal3*) in this same assay (Fig. 1O–U). *Xnr3* is expressed in the organiser as direct response to maternal Wnt signalling (McKendry et al., 1997) and therefore provides a direct readout of canonical Wnt signalling. Consistent with our finding that Sulf1 inhibits axis duplication and ectopic *chordin* expression in response to Wnt8a, we also found that Sulf1 inhibited ectopic *Xnr3* expression in response to Wnt8a (Fig. 1R,T). These results are quantified in Fig. 1U.

### Sulf1 inhibits the ability of Wnt8a to activate the canonical Wnt signalling pathway

**Fig. 2.**
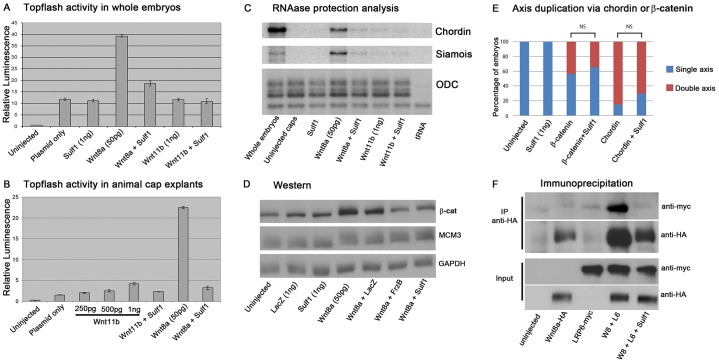
**Sulf1 inhibits the ability of Wnt8a to activate canonical Wnt signalling.** (A) A graph showing the response of the Topflash reporter in whole embryos and (B) in animal caps injected with the mRNAs indicated. Results are mean±s.e.m. (C) An RNase protection analysis shows the expression of *Chordin* and *Siamois* in gastrula stage 10 embryos and in animal caps injected with mRNAs indicated. The expression of *ODC* serves as a loading control, and hybridisation to tRNA controls for specificity. (D) Western blotting for β-catenin shows protein levels in animal caps injected with the mRNAs indicated. Antibodies against MCM and GAPDH serve as loading controls. (E) *Xenopus laevis* embryos were microinjected with mRNA coding for *β-catenin delta-N* (labelled as β-catenin, 150 pg) or *chordin* (150 pg), alone or together with *Sulf1* (1 ng) into a single ventral blastomere at the four-cell stage. The number of embryos with duplicated axes was counted at NF stage 20. (F) Immunoprecipitation of epitope-tagged proteins expressed in animal caps injected with the mRNAs indicated. The top panel shows protein immunoprecipitated with an antibody against HA (Wnt8a is tagged with HA) and immunoblotted with an antibodies against Myc (LRP6 is tagged with Myc). The bottom two panels are the protein lysates prior to immunoprecipitation.

Activation of canonical Wnt signalling results in the stabilisation of β-catenin allowing its accumulation in the nucleus where it forms a complex with Tcf and Lef family proteins to activate gene transcription (Kikuchi et al., 2009; Logan and Nusse, 2004). Topflash is a canonical Wnt reporter that contains Tcf- or Lef-binding sites upstream of the thymidine kinase promoter that drives the expression of luciferase (Molenaar et al., 1996). Overexpression of Wnt8a in whole embryos (Fig. 2A) and in explants (Fig. 2B) resulted in a large increase in Topflash activity, which was inhibited by Sulf1 (Fig. 2A,B). Ectodermal explants from the animal hemisphere of the blastula stage *Xenopus* embryos (‘animal caps’) serve as naïve tissue in which Wnt8a induces the expression of both *chordin* and the direct target of canonical Wnt signalling *siamois*, which were both inhibited by co-expression of Sulf1 (Fig. 2C). Interestingly, Wnt11b had very little activity and did not activate canonical Wnt signalling in these assays whether Sulf1 is present or not (Fig. 2A–C). Overexpression of Wnt8a increased β-catenin protein levels in *Xenopus* animal caps, as analysed by western blotting, whereas the co-injection of mRNAs encoding Sulf1, or the known Wnt antagonist FrzB (also known as Sfrp3), reduced the level of β-catenin detected to similar levels (Fig. 2D). These data demonstrate that Sulf1 can inhibit accumulation of β-catenin and transcriptional output in response to Wnt8a.

HSPGs are required for the activity of many signalling pathways, and it is possible that Sulf1 impacts on other signalling downstream of Wnt to repress axis duplication. We therefore tested whether Sulf1 can affect axis induction by β-catenin or chordin (Fig. 2E). mRNA coding for an activated form of β-catenin (delta-N) (Yost et al., 1996) was injected into a single ventral blastomere at the four-cell stage. Injection of activated β-catenin resulted in 44% of embryos with a duplicated axis, whereas co-expression of Sulf1 with activated β-catenin resulted in 34% with a duplicated axis (a Chi-squared test indicates no significant difference between these values). Similarly, we injected mRNA coding for chordin into a single ventral blastomere at the four-cell stage and found that 85% of the embryos had a duplicated axis, whereas co-injection of Sulf1 and chordin resulted in 70% of embryos with a duplicated axis (no significant difference). These data suggest the effects of Sulf1 on Wnt8a signalling in this assay are direct, and do not reflect effects of Sulf1 on pathways downstream of canonical Wnt signalling.

Sulf1 is secreted and acts extracellularly, and is known to affect receptor–ligand interactions in several signalling pathways (Freeman et al., 2008; Wang et al., 2004). LRP6 is a co-receptor that is required for the stabilisation of β-catenin in response to canonical Wnt signals (Pinson et al., 2000; Tamai et al., 2000; Wehrli et al., 2000). We therefore used immunoprecipitation of tagged LRP6 and Wnt to investigate the effects of Sulf1 on the formation of signalling complexes. LRP6–Myc associated with Wnt8a–HA under normal conditions, as shown by LRP6–Myc being efficiently pulled down with an antibody against HA (Fig. 2F, top panel). This association was disrupted upon co-expression of Sulf1, and LRP6–Myc no longer immunoprecipitated with Wnt8a–HA (Fig. 2F, top panel, right-most lane). These data indicate that Sulf1 acts to disrupt the association of Wnt8a and the co-receptor LRP6, inhibiting the ability of Wnt8a to activate canonical Wnt signalling.

### Sulf1 does not affect Wnt3a-mediated activation of canonical Wnt signalling

**Fig. 3.**
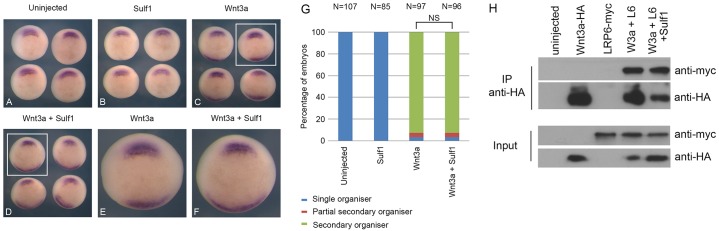
**Sulf1 does not inhibit the ability of Wnt3a to induce axis duplication.** (A–F) *Xenopus laevis* embryos were microinjected with mRNA encoding *Wnt3a* (5 pg) and/or *Sulf1* (1 ng) into a single ventral blastomere at the four-cell stage. *In situ* hybridisation for the gene *chordin* was performed at NF stage 10.5. (A) Uninjected control embryos; (B–D) embryos injected with (B) *Sulf1*, (C) *Wnt3a* or (D) *Sulf1* and *Wnt3a*. The areas indicated in the white boxes in C and D are enlarged in E and F, respectively. (G) The data shown in A–F is quantified in G. NS, not significant (Chi squared test), *N*, number of embryos. (H) Immunoprecipitation (IP) of epitope-tagged proteins expressed in animal caps injected with the mRNAs indicated. The top panel shows protein immunoprecipitated with an antibody against HA (Wnt3a is tagged with HA) and immunoblotted with an antibodies against Myc (LRP6 is tagged with Myc). The bottom two panels are protein lysates prior to immunoprecipitation.

As shown above, Sulf1 has the ability to inhibit Wnt8a-mediated activation of canonical Wnt signalling in *Xenopus*, and we used similar methods to analyse the effects of Sulf1 on Wnt3a activity. Like Wnt8a, Wnt3a is defined as a canonical Wnt ligand and induces axis duplication when overexpressed in *Xenopus* (Du et al., 1995; Wolda et al., 1993). Overexpression of Wnt3a in a single ventral blastomere at the four-cell stage induced an ectopic region of chordin expression (Fig. 3C), similar to that shown for Wnt8a (Fig. 1). However, overexpression of Sulf1 together with Wnt3a did not inhibit the ability of Wnt3a to induce ectopic chordin expression (Fig. 3D,F). The data in Fig. 3A–F is quantified in Fig. 3G. We conclude that Sulf1 does not inhibit the activity of all canonical Wnts. To test the possibility that Sulf1 enhances Wnt3a activity in *Xenopus*, low levels of *Wnt3a* mRNA were injected alone or together with Sulf1, but no change in the frequency of ectopic *chordin* expression was found (supplementary material Fig. S1). We used the same LRP6 immunoprecipitation assay as for Wnt8a, and found that LRP6–Myc associated with Wnt3a–HA when expressed in *Xenopus*. LRP6–Myc was efficiently pulled down with an antibody against HA (Fig. 3H, top panel). In contrast to our results with Wnt8a, there was no effect on this association when Sulf1 was co-expressed. These data indicate that Sulf1 is not a universal inhibitor of canonical Wnt signalling in *Xenopus*, but rather has ligand-specific effects.

### Sulf1 enhances the ability of Wnt11b to activate canonical and non-canonical Wnt signalling

**Fig. 4.**
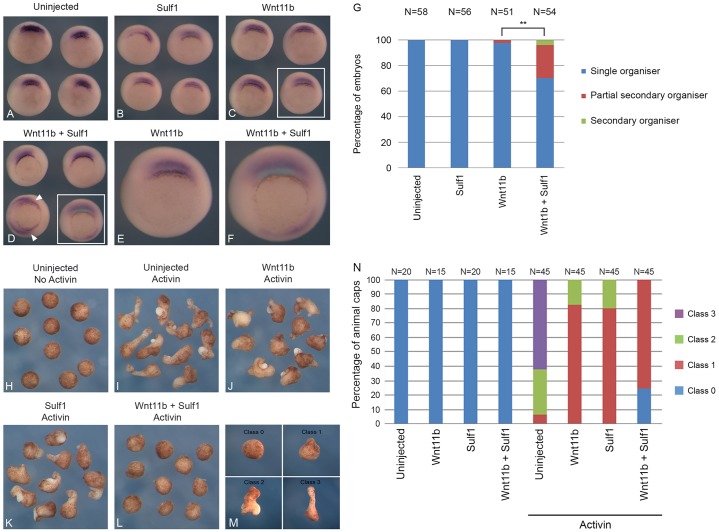
**Sulf1 enhances the ability of Wnt11b to activate both canonical and non-canonical Wnt signalling.** (A–F) *Xenopus laevis* embryos were microinjected with mRNA encoding *Wnt11b* (600 pg) and/or *Sulf1* (1 ng) into a single ventral blastomere at the four-cell stage. *In situ* hybridisation for the gene *chordin* was performed at NF stage 10.5. (A) Uninjected control embryos; (B–D) embryos injected with (B) *Sulf1*, (C) *Wnt3a* or (D) *Sulf1* and *Wnt11b*. The areas indicated in the white boxes in in C and D are enlarged in E and F, respectively. (G) The data in A–F is quantified in G. **P<0.01 (Chi squared test). (H–L) Embryos were microinjected bilaterally in the animal hemisphere with mRNA encoding *Wnt11b* (50 pg) and *Sulf1* (500 pg). Embryos were cultured until NF stage 8, animal explants were taken and cultured until NF stage 10.5 in either the presence or absence of activin. (H,I) Control animal explants culture in either the absence (H) or presence (I) of activin. (J–L) Animal explants injected with (J) *Wnt11b*, (K) *Sulf1* or (L) *Wnt11b* and *Sulf1*. (M) The classification system used to score animal cap explants shown in N. (N) The data shown in H–L is quantified in N. *N*, number of embryos.

Our previous work (Freeman et al., 2008) has demonstrated that overexpression of Sulf1 enhances the ability of the non-canonical Wnt ligand, *Xenopus tropicalis* Wnt11b2 (Gilchrist et al., 2004) to activate canonical Wnt signalling. Here, we therefore tested whether Sulf1 has a similar effect on *Xenopus laevis* Wnt11b (Ku and Melton, 1993). mRNA encoding Sulf1 and Wnt11b were microinjected into a single ventral blastomere at the four-cell stage and assayed for *chordin* expression. Overexpression of Wnt11b did not induce ectopic *chordin* expression (Fig. 4C). However, when Sulf1 was overexpressed together with Wnt11b, some induction of ectopic *chordin* expression was detected (about 30%; Fig. 4D), as well as some partial axis duplication at stage 36 (data not shown). The data in Fig. 4A–F are quantified in Fig. 4G, and indicate that Sulf1 can moderately enhance the ability of Wnt11b to activate canonical Wnt signalling in *Xenopus*. It is interesting to note that maternal mRNAs coding for Wnt11b and Sulf1 colocalise in the dorsal vegetal region of cleavage stage embryos (Freeman et al., 2008), suggesting an interaction. However, in ectodermal explants, overexpression of Wnt11b on its own or together with Sulf1 does not activate canonical Wnt signalling (Fig. 2A–C).

Wnt11b is required to regulate medial and lateral convergent extension during zebrafish gastrulation (Heisenberg et al., 2000). In *Xenopus*, convergent extension cell behaviour can be elicited by treating animal cap explants with the TGFβ signalling molecule activin. Activin induces the formation of dorsal mesoderm and, as a result, the cells undertake the same convergent extension cell behaviour as seen in the dorsal mesoderm during gastrulation (Asashima et al., 1990; Smith, 1987). Untreated animal caps formed atypical ciliated epidermis and remained round (Fig. 4H), whereas explants treated with activin formed elongated structures that contained muscle and notochord (Fig. 4I). Inhibition or over-activation of non-canonical Wnt signalling is known to inhibit convergent extension because in either case cell polarity is lost (Tada and Smith, 2000; Wallingford et al., 2002; Wallingford and Harland, 2001; Wallingford et al., 2000). Overexpression of either Wnt11b or Sulf1 reduced activin-induced convergent extension in animal cap explants (Fig. 4J,K) and their co-expression enhanced this effect, resulting in the complete inhibition of convergent extension (Fig. 4L). Quantification of convergent extension was performed using the classification system shown in Fig. 4M, and the results are quantified in Fig. 4N, and indicate that Sulf1 might synergise with Wnt11b to inhibit activin-induced convergent extension.

### Sulf1 enhances Wnt11b induced Dvl–GFP translocation to the plasma membrane

Either the activation or inhibition of Wnt11b signalling disrupts convergent extension during gastrulation (Tada and Smith, 2000), and fibroblast growth factor (FGF) signalling is also required for this cell behaviour (Cornell and Kimelman, 1994). To investigate whether the effect of Sulf1 expression on explant elongation is due to an inhibition or activation of Wnt11b-mediated non-canonical Wnt signalling, or effects on FGF signalling, we measured the subcellular localisation of a Dvl–GFP fusion protein (Yang-Snyder et al., 1996). Activation of non-canonical Wnt signalling results in the translocation of Dvl–GFP to the plasma membrane (Miller et al., 1999; Rothbächer et al., 2000; Yamanaka and Nishida, 2007), whereas inhibition of FGF signalling prevents it (Shi et al., 2009).

**Fig. 5.**
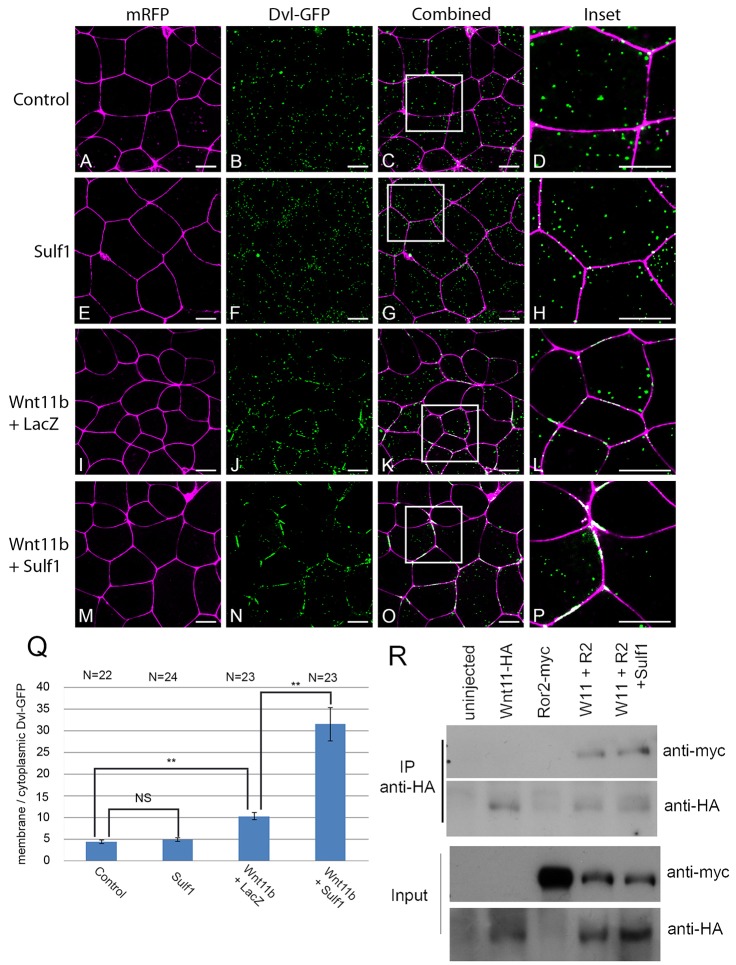
**Sulf1 enhances Wnt11b induced Dvl–GFP translocation to the cell membrane.** (A–P) *Xenopus laevis* embryos were microinjected bilaterally with mRNA encoding *mRFP* (500 pg) and *Dvl–GFP* (500 pg) into the animal hemisphere at the two-cell stage. In addition embryos were injected with mRNA encoding *Sulf1* (4 ng), *LacZ* (4 ng), *Wnt11b* (400 pg) or a mixture of the three. (A–D) Control animal explants overexpressing mRFP and Dvl–GFP. Animal explants injected with (E–H) *Sulf1*, (I–L) *Wnt11b* and *LacZ* or (M–P) *Sulf1* and *Wnt11b*. The white boxes in C, G, K and O mark the areas that are enlarged in D, H, L and P, respectively. mRFP is shown in magenta, Dvl–GFP is shown in green. Scale bars: 20 μm. (Q) The data shown in A–P is quantified in Q. Quantification was done using a program written in MatLab, results are mean±s.e.m. **P<0.01; NS, not significant (Mann–Whitney U test). *N*, number of embryos. (R) Immunoprecipitation (IP) of epitope-tagged proteins expressed in animal caps injected with the mRNAs indicated. The top panel shows protein immunoprecipitated with an antibody against HA (Wnt11b is tagged with HA) and immunoblotted with an antibodies against Myc (Ror2 is tagged with Myc). The bottom two panels are protein lysates prior to immunoprecipitation.

Embryos were microinjected with mRNA encoding mRFP and Dvl–GFP, and at blastula stage 8 animal cap explants were analysed by confocal microscopy. In control conditions, Dvl–GFP was visualised as discrete puncta in the cytoplasm (Fig. 5A–D), whereas mRFP highlights the plasma membrane. Overexpression of Sulf1 had no effect on the subcellular localisation of Dvl–GFP (Fig. 5E–H). Overexpression of Wnt11b signalling induced the translocation of Dvl–GFP to the plasma membrane where it formed aggregates (Fig. 5I–L). Increasing the amount of Wnt11b mRNA injected from 400 to 800 pg enhanced the accumulation of Dvl–GFP on the cell membrane (supplementary material Fig. S2). Overexpression of Sulf1 together with Wnt11b significantly enhanced the accumulation of Dvl–GFP on the plasma membrane, with very little Dvl–GFP remaining in the cytoplasm and Dvl–GFP forming thick aggregates on the cell membrane (Fig. 5M–P). The amount of Dvl–GFP colocalised with the plasma membrane was calculated relative to the amount of cytoplasmic Dvl–GFP over several experimental samples (Fig. 5Q). These data indicate that Sulf1 significantly increases Wnt11b induction of non-canonical Wnt signalling, as measured by membrane translocation of Dvl. In addition, Sulf1 enhanced the ability of Wnt11b to activate the non-canonical Wnt luciferase reporter ATF2 (Ohkawara and Niehrs, 2011) (supplementary material Fig. S2). In contrast, we found no membrane accumulation of Dvl–GFP in response to Wnt8a, and coexpression of Sulf1 did not change this; however, Sulf1 did enhance membrane accumulation of Dvl–GFP in response to Wnt4 (data not shown).

To investigate any ability of Sulf1 to alter the assembly of non-canonical Wnt signalling complexes, we used immunoprecipitation of a Myc-tagged Ror2, a known co-receptor for the non-canonical Wnt pathway (Hikasa et al., 2002). We found that Ror2–Myc associated with Wnt11b–HA when expressed in *Xenopus*, and that Ror2–Myc was efficiently pulled down with an antibody against HA (Fig. 5R, top panel). This association was not changed when Sulf1 was co-expressed, and Ror2–Myc immunoprecipitated with Wnt11b–HA at similar levels (Fig. 5R, top panel, right-most lane). These data indicate that although Sulf1 can dramatically enhance non-canonical Wnt signalling in response to Wnt11b, it does not act through increasing or decreasing the association of Wnt11b with its co-receptor Ror2. This indicates that Sulf1 can act through distinct molecular mechanisms to impact on Wnt signalling.

### Sulf1 enhances the accumulation of Wnt11b–GFP on the cell membrane

**Fig. 6.**
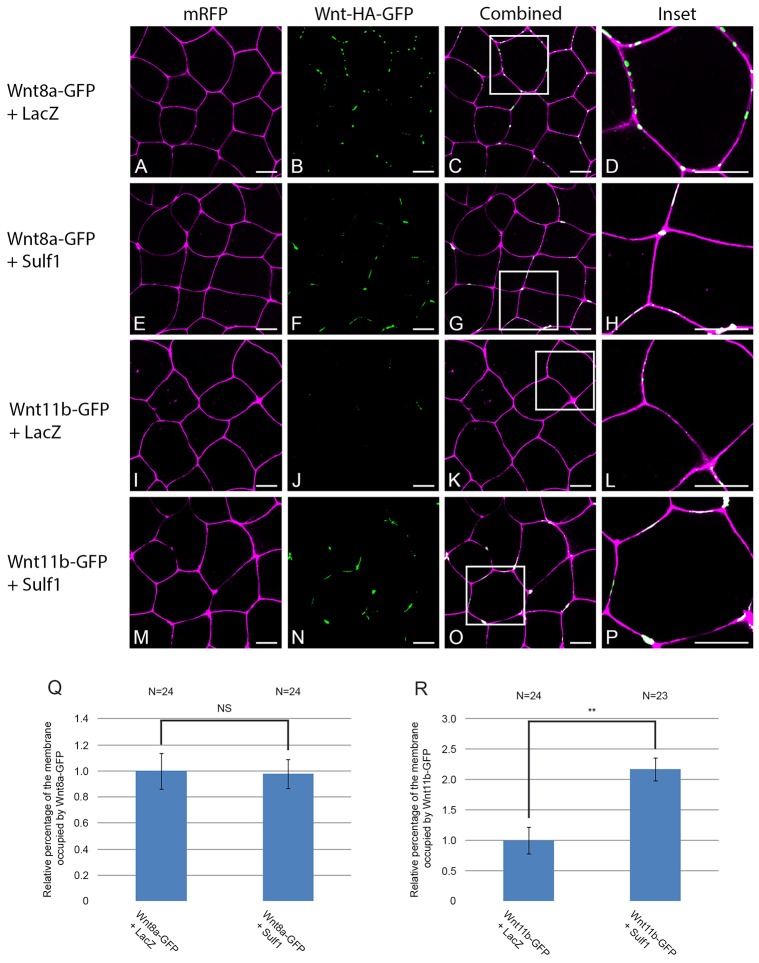
**Sulf1 enhances the accumulation of Wnt11b–GFP on the cell membrane.** (A–P) *Xenopus laevis* embryos were microinjected bilaterally with mRNA encoding *mRFP* (500 pg) into the animal hemisphere at the two-cell stage. In addition, embryos were injected with mRNA encoding *LacZ* (4 ng), *Sulf1* (4 ng), *Wnt8a–GFP* (500 pg), *Wnt11b–GFP* (1 ng) or a mixture of the four. (A–D) Control animal explants overexpressing LacZ and Wnt8a–GFP. (E–H) Animal explants overexpressing Sulf1 and Wnt8a–GFP. (I–L) Control animal explants overexpressing LacZ and Wnt11b–GFP. (M–P) Animal explants overexpressing Sulf1 and Wnt11b–GFP. The white boxes in C, G, K and O mark the areas that are enlarged in D, H, L and P, respectively. mRFP is shown in magenta, Wnt8a– or Wnt11b–GFP is shown in green. Scale bars: 20 μm. (Q,R) Graphs quantifying the relative levels of (Q) Wnt8a–GFP and (R) Wnt11b–GFP on the cell membrane. Data was quantified using a programme written in Matlab, results are mean±s.e.m. **P<0.01; NS, not significant (Mann–Whitney U test). *N*, number of embryos.

Experiments using *Drosophila* have shown that Sulf can regulate the extracellular distribution of Wg in the developing wing disc (Kleinschmit et al., 2010; You et al., 2011). To examine whether Sulf1 could regulate the cell surface localisation or diffusion of Wnt ligands in *Xenopus*, we generated the GFP fusion proteins Wnt8a–GFP and Wnt11b–GFP, which retain biological activity when overexpressed (supplementary material Fig. S3). After injection of mRNA coding for Wnt8a– or Wnt11b–GFP together with mRFP into the animal hemisphere of *Xenopus* embryos, animal cap explants were analysed by confocal microscopy. Wnt8a–GFP was visible as discrete puncta, which colocalised with the cell membrane (Fig. 6A–D), and the amount of Wnt8a present at the membrane was not affected by co-expression of Sulf1 (Fig. 6E–H; quantified in Fig. 6Q). In contrast, very little Wnt11b–GFP expression could be seen on the cell membrane in control conditions (Fig. 6I–L). This is not due to a lack of protein expression as Wnt11b–GFP could be detected by western blot in animal caps (supplementary material Fig. S4). Overexpression of Sulf1 dramatically enhanced the levels of Wnt11b–GFP colocalising with the cell membrane (Fig. 6M–P; quantified in Fig. 6R).

### Sulf1 enhances the range of Wnt8a–GFP diffusion in animal cap explants

**Fig. 7.**
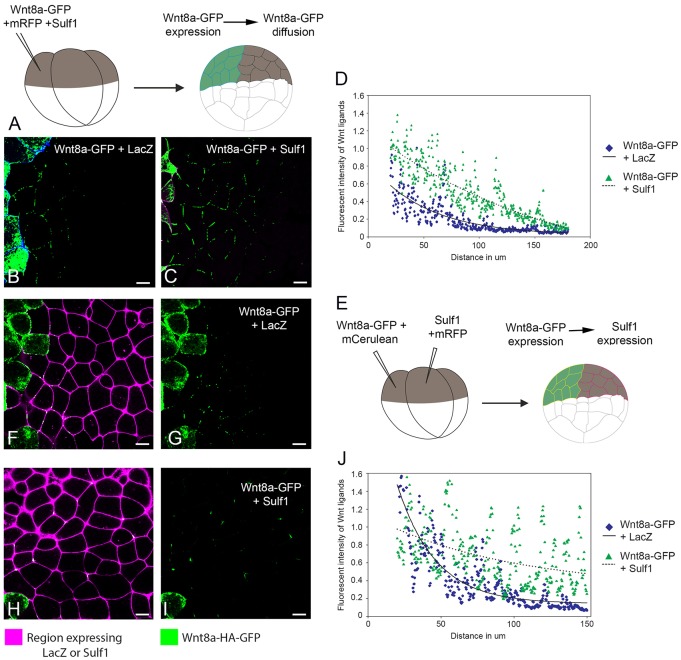
**Sulf1 enhances the secretion and range of diffusion of Wnt8a–GFP in animal explants.** (A) Diagram depicting the assay used to measure Wnt8a–GFP secretion and diffusion through a control background, see Materials and Methods for details. (B,C) mRNA encoding either (B) *mCerulean* (600 pg), *LacZ* (4 ng) and *Wnt8a–GFP* (2 ng) or (C) *mRFP* (600 pg), *Sulf1* (4 ng) and *Wnt8a–GFP* (2 ng) was injected into the animal hemisphere of one blastomere at the four-cell stage. (D) The range of diffusion of Wnt8a–GFP through a control background was quantified using Fiji Image J. (E) Diagram depicting the assay used to measure Wnt8a–GFP diffusion through a background expressing Sulf1. (F–I) mRNA encoding *mCerulean* (600 pg) and *Wnt8a–GFP* (2 ng) was injected into the animal hemisphere of one blastomere at the four-cell stage. An adjacent blastomere was injected with mRNA encoding (F,G) *mRFP* (600 pg) and *LacZ* (4 ng) or (H,I) *mRFP* (600 pg) and *Sulf1* (4 ng). (J) The range of Wnt8a–GFP through a background expressing either LacZ or Sulf1 was quantified using Fiji Image J. Scale bars: 20 μm.

We tested the ability of Sulf1 to influence Wnt8a diffusion when co-expressed in the ligand-producing cell. At the four-cell stage, a single animal blastomere was microinjected with mRNAs encoding Wnt8a–GFP together with a membrane marker and either Sulf1 or LacZ (as a control) (Fig. 7A). The level of GFP fluorescence was measured across the field of cells, with mCerulean marking the source of the fluorescent Wnt ligand. We found that in control conditions, Wnt8a–GFP was capable of diffusing two or three cell diameters away from the cells expressing it (Fig. 7B). Co-expression of Sulf1 significantly increased the range of Wnt8a–GFP diffusion to ∼six or seven cell diameters (Fig. 7C). The effects of Sulf1 on Wnt8a–GFP diffusion for multiple experiments were quantified (Fig. 7D). The graph shows the reduction in the average fluorescence intensity of Wnt8a–GFP with increasing distance from Wnt secreting cells. Sulf1 increased the levels of Wnt8a–GFP further from the source as compared to controls (Fig. 7D). The lines of best fit for the Wnt8a–GFP distribution show similar rates of decay under both control and Sulf1 conditions. One prediction from this is that Sulf1 does not alter the qualitative nature of Wnt8a–GFP diffusion, but rather enhances the levels of Wnt8a–GFP released from Wnt-secreting cells, allowing increased range of signalling.

In another experiment, we tested the ability of Sulf1 to influence the diffusion of Wnt8a when it is expressed in the ligand-receiving cells: mRNAs coding for Wnt8a–GFP and Sulf1 were injected into adjacent animal blastomeres at the four-cell stage (see Fig. 7E) along with lineage tracers. We found that under control conditions, Wnt8a–GFP diffused two or three cell diameters (Fig. 7F,G). However, Wnt8a–GFP was able to diffuse a greater distance through a region overexpressing Sulf1 (Fig. 7H–J). In addition, regression analysis indicated that the rate of decay of Wnt8a–GFP signal intensity was ∼3.5 times less when diffusing through a region expressing Sulf1 in comparison to LacZ, suggesting that overexpression of Sulf1 reduces the normal rate of decay of Wnt8a–GFP resulting in a more uniform distribution across the field of Sulf1-overexpressing cells, as compared to the rapid reduction of fluorescence intensity seen in control conditions (see Fig. 7J). These data indicate that Sulf1, whether expressed in Wnt secreting or receiving cells, can increase the range of Wnt8a–GFP diffusion.

### Sulf1 enhances both the levels and the range of Wnt11b–GFP diffusion in animal cap explants

**Fig. 8.**
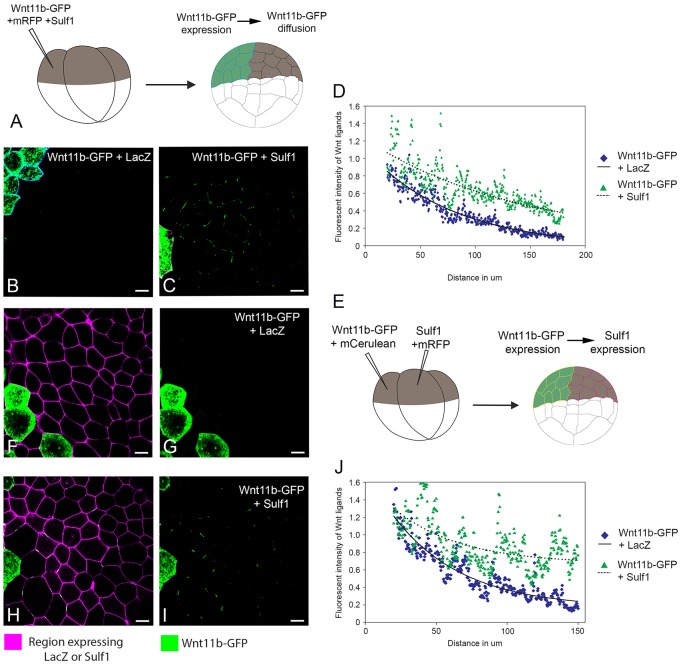
**Sulf1 enhances both the secretion and the amount of Wnt11b–GFP diffusing in animal explants.** (A) Diagram depicting the assay used to measure Wnt11b–GFP secretion and diffusion through a control background. (B,C) mRNA encoding either (B) *mCerulean* (600 pg), *LacZ* (4 ng) and *Wnt11b-GFP* (2 ng) or (C) *mRFP* (600 pg), *Sulf1* (4 ng) and *Wnt11b–GFP* (2 ng) was injected into the animal hemisphere of one blastomere at the four-cell stage. (D) The range of diffusion of Wnt11b–GFP through a control background was quantified using Fiji Image J. (E) Diagram depicting the assay used to measure Wnt11b–GFP diffusion through a background expressing Sulf1. (F–I) mRNA encoding *mCerulean* (600 pg) and *Wnt11b–GFP* (2 ng) was injected into the animal hemisphere of one blastomere at the four-cell stage. An adjacent blastomere was injected with mRNA encoding (F,G) *mRFP* (600 pg) and *LacZ* (4 ng) or (H,I) *mRFP* (600 pg) and *Sulf1* (4 ng). (J) The range of Wnt11b–GFP through a background expressing either *LacZ* or *Sulf1* was quantified using Fiji Image J. Scale bars: 20 μm.

Using the approach described above (Fig. 8A), we tested the ability of Sulf1 to influence Wnt11b diffusion when co-expressed in the ligand-producing cell. We found that, in contrast to Wnt8a–GFP, very little Wnt11b–GFP could be detected in explants co-injected with control mRNA coding for LacZ (Fig. 8B). Overexpression of Sulf1 together with Wnt11b–GFP dramatically enhanced the presence of Wnt11b–GFP at the cell surface and across the field of cells (Fig. 8C). Similar to its effects on Wnt8a–GFP, Sulf1 enhanced the levels of Wnt11b–GFP at a distance from the source; however, Sulf1 did not alter the rate of decay of Wnt11b–GFP fluorescence intensity (Fig. 8D). One interpretation of this result is that Sulf1 can increase the overall levels of Wnt11b outside the cell, without changing the qualitative nature of its diffusion.

To test the ability of Sulf1 to influence the diffusion of Wnt11b when it is expressed in the ligand-receiving cells, we injected mRNAs coding for Wnt11b–GFP and Sulf1 into adjacent animal blastomeres at the four-cell stage (see Fig. 8E) along with lineage tracers. Very little Wnt11b–GFP could be detected in control explants (Fig. 8F,G); however, the presence of Sulf1 resulted in a dramatic increase in fluorescence. This suggests that Wnt11b–GFP is able to diffuse much further through a region overexpressing Sulf1 (Fig. 8H,I). Regression analysis indicates there is little or no change in the rate of decay of Wnt11b–GFP fluorescence intensity with increasing distance from the source when compared with control conditions (Fig. 8J). This steady reduction in Wnt11b–GFP fluorescence intensity when diffusing though a region expressing Sulf1 contrasts with the lower rate of signal decay observed for Wnt8a–GFP (compare Fig. 8J with Fig. 7J). Our interpretation of these data is that Sulf1 can extend the range of Wnt8a diffusion, and by doing so, alter the shape of the Wnt8a gradient. In contrast, Sulf1 extends the range of Wnt11b diffusion by increasing the overall levels of Wnt11b present on the cell membrane. The outcome of this would be an increased amount of Wnt11b available to cells in the presence of Sulf1, while the overall shape of the morphogen gradient is preserved; this effect could underpin the ability of Sulf1 to enhance non-canonical Wnt signalling.

## DISCUSSION

Sulf enzymes have been recognised for their ability to enhance canonical Wnt signalling; indeed Sulf1 was first identified as a positive regulator of Wnt-mediated MyoD activation in the quail somite (Dhoot et al., 2001). In assays using reconstituted cell cultures, Sulf1 has been found to enhance the abilities of Wnt1 (Ai et al., 2003; Dhoot et al., 2001; Hitchins et al., 2013; Nawroth et al., 2007), Wnt3, Wnt3a (Tang and Rosen, 2009), Wnt4 (Nawroth et al., 2007) and Wnt6 (Hitchins et al., 2013) to activate canonical Wnt signalling. Increased expression of Sulf1 and Sulf2 in some cancers, including pancreatic adenocarcinomas (Nawroth et al., 2007) and non-small cell lung cancers (Lemjabbar-Alaoui et al., 2010) is associated with overactive Wnt signalling. The ability to reduce canonical Wnt signalling and reverse the transformed phenotypes by knocking down Sulf expression in these cell lines lends further weight to the notion that Sulf enzymes positively regulate canonical Wnt signalling (Lemjabbar-Alaoui et al., 2010; Nawroth et al., 2007; Rosen and Lemjabbar-Alaoui, 2010). Our data, however, demonstrates that the effects of Sulf1 on distinct Wnt ligands are different depending on cellular context.

Over the last twenty years, *Xenopus* has been central in deciphering many aspects of vertebrate Wnt signalling (Hoppler and Moon, 2014) including the identification of the low-density lipoprotein (LDL)-related co-receptors (Tamai et al., 2000), the role of β-catenin and TCF proteins in axis induction (Molenaar et al., 1996), the discovery of secreted frizzled receptors like FzB that antagonise Wnt signalling (Leyns et al., 1997; Wang et al., 1997), and indeed, even the initial description of the canonical and non-canonical pathways was elucidated in *Xenopus* (Du et al., 1995). This study takes advantage of this well-established system to analyse the impact of Sulf1 activity on both canonical and non-canonical Wnt signalling.

Sulf1 is expressed maternally in *Xenopus* and transcripts colocalise in the oocyte with those encoding Wnt11b (Freeman et al., 2008), a Wnt ligand shown to be essential for dorsal axis specification (Tao et al., 2005). The zygotic expression of Sulf1 is dynamic and overlaps at times with that of Wnt ligands, such as with Wnt11b and Wnt8a, in the posterior mesoderm and later with Wnt4 in the pronephros. Antisense morpholino knockdown of Sulf1 in *Xenopus* (Freeman et al., 2008) results in a phenotype consistent with overactive Wnt signalling (Fredieu et al., 1997); however, similar defects result from increases in FGF (Pownall et al., 1996) or BMP (Hartley et al., 2001) signalling, both of which are affected by loss of Sulf1 (Meyers et al., 2013). In this paper, we examine the activity of Sulf1 in the context of specific Wnt ligands and their downstream cellular responses.

### Cell-autonomous and non-cell-autonomous effects of Sulf1

Our novel finding that Sulf1 inhibits the ability of Wnt8a to induce a second axis in *Xenopus* embryos was followed up with several other experiments to corroborate our results. These data show that Sulf1 consistently inhibits the ability of Wnt8a to stabilize β-catenin, stimulate Topflash activity or induce the expression of *chordin*, *Xnr3* or *siamois*. The ability of Sulf1 to prevent the association of Wnt8a with LRP6 suggests that there is a mechanism where Sulf1-modified heparan sulfate is not compatible with the formation or maintenance of the receptor–ligand signalling complex necessary for canonical Wnt signalling (He et al., 2004). From this, we conclude that Sulf1 activity inhibits Wnt8a in a cell-autonomous manner by obstructing signalling at the cell surface.

In keeping with our findings, there is other evidence in the literature that the Sulf enzymes do not always enhance canonical Wnt signalling. Human Sulf1 is silenced in the gastric cancer cell line MKN28, and when Sulf1 expression is restored, the oncogenic phenotype is reduced along with a reduction in canonical Wnt signalling (Li et al., 2011). The ability of Sulf1 to inhibit the stabilization of β-catenin when re-expressed in gastric tumours indicates that, in this context, Sulf1 inhibits canonical Wnt signalling. *Drosophila* mutants deficient in Sulf display a wing phenotype consistent with elevated Wg signalling (Kleinschmit et al., 2010; You et al., 2011), indicating that the normal role of Sulf is to negatively regulate Wg.

During development, Sulf^−/−^ flies display increased levels of extracellular Wg protein in the wing disc and a steeper gradient of Wg from the dorsal-ventral margin, resulting in a disruption of the normal Wg morphogen gradient (Kleinschmit et al., 2010). The requirement for Sulf in shaping the Wg morphogen in flies suggests that there might be alternative molecular mechanisms by which Sulf enzymes modulate Wnt signalling in addition to the widely accepted ‘catch and present’ model (Ai et al., 2003). Sulf has been shown to also modify the hedgehog morphogen in both *Drosophila* (Wojcinski et al., 2011) and vertebrate embryos (Danesin et al., 2006; Ramsbottom et al., 2014; Touahri et al., 2012).

Sulf1 can influence the extracellular distribution of Wnt ligands in *Xenopus* explants such that Sulf overexpression imparts a larger range of Wnt8a diffusion across a field of cells. A close examination of *chordin* expression resulting from Sulf1 and Wnt8a co-expression in a single ventral blastomere (Fig. 1M) reveals small wings of *chordin* expression that appear outside the region induced by ectopic Wnt8a signalling in controls. We propose that Sulf1 inhibits Wnt8a activation of *chordin* expression in the cells that express the injected Sulf1 in a cell-autonomous way by disrupting the association of the LRP6 signalling complex. We also suggest that the protein produced from the injected *Wnt8a* mRNA is able to spread further in the presence of Sulf1 and therefore can activate *chordin* expression in adjacent cells not expressing Sulf1. These findings support the notion that Sulf1 can impact on Wnt signals in distinct ways, affecting both the spread of Wnt ligands and their reception at the cell surface.

### Context dependent, ligand dependent effects of Sulf1 on canonical Wnt signalling

Although we show that Sulf1 inhibits Wnt8a signalling, we find, however, that Sulf1 is not a global inhibitor of canonical Wnt signalling (Ai et al., 2003). Indeed, we show that Sulf1 has no detectable effect on Wnt3a activity in our assays; Sulf1 neither inhibits (Fig. 3) nor enhances (supplementary material Fig. S1) axis induction by Wnt3a, nor does it affect the association of Wnt3a with LRP6 (Fig. 3H). In contrast to Sulf1 inhibition of Wnt8a, we show here and previously (Freeman et al., 2008) that ventral overexpression of Sulf1 with Wnt11b elicits some axis-inducing activity. Although widely accepted as a non-canonical Wnt, Wnt11b has been shown to stimulate the canonical pathway. It has been shown that maternal depletion of Wnt11b results in the loss of a dorsal axis (a structure that requires maternal canonical Wnt signalling) which can be rescued by overexpressing β-catenin (Tao et al., 2005). Interestingly, this same work also showed a requirement for the heparan sulfate polymerase EXT1 in axis specification. Transcripts for Wnt11b and Sulf1 colocalise in the *Xenopus* oocyte (Freeman et al., 2008), and we speculate that maternal Sulf1 might have a role in endogenous axis specification by enhancing the ability of maternal Wnt11b to activate canonical Wnt signalling. Confirmation of this hypothesis will require depletion of maternal mRNAs such as shown previously (Tao et al., 2005), or other genetic approaches.

### Sulf1 enhances the level and range of Wnt11b ligand and its downstream signalling

Sulf1 enhances the activity of both Wnt11b and Wnt4 (data not shown) in assays for non-canonical Wnt signalling. Sulf1 expression induces quantitative changes in the amount of Wnt11b–GFP visible on the cell surface (there is more), as well as qualitative changes in the size and shape of Wnt11b–GFP puncta (they are larger and longer). The increased levels of Wnt11b result in a greater range for the ligand in the presence of Sulf1; however, there is no change in the rate of decay (Fig. 8). The enhancement of non-canonical Wnt signalling in the presence of Sulf1 might simply reflect an increase in the stability of Wnt ligand and its availability on the cell surface; unlike with Wnt8a–LRP6, we find no change in the affinity of the Ror2 co-receptor for Wnt11b in the presence of Sulf1. The enhancement of non-canonical Wnt signalling by Sulf enzymes is not universal; cultured myoblasts deficient for both Sulf1 and Sulf2 exhibit higher levels of Ca^2+^/calmodulin-dependent protein kinase II (CaMKII) than controls, and newly formed myofibres have fewer nuclei, suggesting that Sulf1 is required to restrict non-canonical Wnt signalling in myoblasts to ensure sufficient numbers of cells are present prior to fusion (Tran et al., 2012).

### Perspectives on Sulf1 regulation of cell signalling

Any biological effect of Sulf1 needs be considered in light of all signalling pathways that require HSPGs, and the FGF pathway is of particular importance when considering non-canonical Wnt signalling. Like non-canonical Wnt signalling, FGF signalling is also required for convergent extension in *Xenopus* embryos and explants (Amaya et al., 1991; Cornell and Kimelman, 1994; Isaacs et al., 1994). These overlapping outputs of FGF and non-canonical Wnt signalling make it challenging to decipher the target of Sulf1 action, as Sulf1 is a potent inhibitor of FGF signalling in *Xenopus* development and cell culture (Freeman et al., 2008; Lamanna et al., 2008; Wang et al., 2004). Dysregulation of FGF or Wnt signalling is the basis of some cancers (Clevers, 2006; Knights and Cook, 2010), and consistent with a role for Sulf enzymes in these pathways, human *SULF1* and *SULF2* are also mis-expressed in cancer (Rosen and Lemjabbar-Alaoui, 2010; Vivès et al., 2014). An overview of SULF gene expression in human cancers indicates that although *SULF1* is sometimes downregulated (Khurana et al., 2013), more often *SULF1* and/or *SULF2* are overexpressed in tumours, which can be associated with a worse prognosis (Lemjabbar-Alaoui et al., 2010). A role for Sulfs in regulating non-canonical Wnt signalling might be relevant to cancer progression, as planar cell polarity (PCP) signalling has been implicated in metastasis and cell migration (Mayor and Theveneau, 2014; Nishita et al., 2010), and, consistent with our data, these effects are context dependent where the cellular response to non-canonical Wnt signal depends on tumour type (MacMillan et al., 2014). The fact that SULFs are extracellular enzymes makes them attractive targets for the development of new drugs or diagnostics. A better understanding of the molecular and cellular mechanisms underlying Sulf activity and will inform and advance these efforts.

## MATERIALS AND METHODS

### Plasmids

Plasmids used to generate synthetic mRNAs for microinjection have been published previously: Wnt11b (Tao et al., 2005), Wnt8a (Christian et al., 1991), Wnt3a (Faas and Isaacs, 2009), Sulf1 (Freeman et al., 2008), Chordin (Piccolo et al., 1997), activated β-catenin (Yost et al., 1996), LRP6–Myc (Tamai et al., 2000), and pBluescript(RN3) Dvl-GFP (Yang-Snyder et al., 1996). The plasmids coding for the fluorescent Wnt ligands were constructed as follows: Wnt8a and Wnt11b were sub-cloned through PCR into pCS2+ using Phusion (New England BioLabs) where the reverse primers include sequence for an HA tag and removed the endogenous stop codons. Subsequently GFP was sub-cloned onto the C-terminus of the HA tag, resulting in plasmids coding for Wnt–HA–GFP fusion proteins that were used in this study.

### Injections

mRNAs for injection were synthesised using SP6 megascript kit (Ambion), except Dvl–GFP which was synthesised using the T3 megascript kit (Ambion); the amount of mRNA injected is indicated for each experiment in the Results section.

### Ribonuclease protection assay

mRNAs coding for XWnt8a (50 pg), XWnt11b (1 ng) and Sulf1 (4 ng) were injected into two-cell stage *Xenopus laevis* embryos and animal cap explants were dissected at stage 8 and cultured until stage 13. RNA was extracted in NETS (300 mM NaCl, 10 mM Tris-HCl pH 8, 1 mM EDTA, 5% SDS) followed by phenol-chloroform extraction and ethanol precipitation. RNase protection analyses were performed as described previously (Isaacs et al., 1994) with a hybridisation temperature of 45°C. P32-labelled RNA probes for *chordin*, *siamois* and the loading control *ornithine* d*ecarboxylase* (*ODC*) were prepared as described previously (Isaacs et al., 1992).

### *In situ* hybridisation

Embryos were fixed in MEMFA (0.1 M MOPS, 2 mM EDTA, l mM MgSO_4_, 3.7% formaldehyde) for l hour at room temperature. *In situ* hybridization was carried out as modified from Harland (Harland, 1991) and detailed previously (Fisher et al., 2003). Probes for *in situ* hybridisation were transcribed using l0× DIG RNA labelling mix (Roche) from linearised plasmids for *chordin* and Xnr3 (IMAGE: 5161617 and IMAGE: 7297499).

### Dual luciferase assay

Two-cell embryos were co-injected with 10 pg TopFlash (Invitrogen) and 1 pg Renilla CMV (Invitrogen) plasmids together with mRNAs coding for XWnt8a (50 pg), XWnt11b (0.25–1 ng), with or without Sulf1 (4 ng). At NF stage 8.5, animal caps were dissected and cultured until stage 10, snap frozen on dry ice and analysed using the Dual Luciferase kit (Promega) as directed by the manufacturer. Three sample sets were analysed per condition and comparisons were made using a two-tailed Student's *t*-test. A Lumat LB 9501 luminometer (Berthoid) was used to measure luminescent activity and relative luminescent units were determined by dividing the luciferase by the *Renilla* values. Plasmid DNA encoding 100 pg of the ATF reporter (Ohkawara and Niehrs, 2011) and Renilla-TK plasmid DNA was injected into the marginal zone of all four cells of a four-cell stage embryo along with the indicated mRNAs. The luciferase assay was performed using five whole embryos per reaction and carried out using the dual luciferase reporter assay system kit.

### Animal cap assays

To analyse convergent extension, *Xenopus laevis* embryos were injected at the two-cell stage with the indicated mRNAs. Animal caps were isolated at stage 8 and cultured until stage 10.5 and frozen for western blotting, or until stage 19 in either the presence or absence of activin to analyse convergent extension. For confocal microscopy, *Xenopus* embryos were either injected at the two-cell stage or at the stage indicated in the results, after which animal caps were isolated at stage 8 and then cultured in the dark for 8 hours at 21°C. For confocal microscopy, animal caps were mounted on relief slides, which were generated by coating slides with two layers of PVC insulation tape and then cutting a 10×14 mm hole in the tape. Confocal microscopy was carried out using the inverted laser scanning microscope LSM710 (Carl Zeiss) and Zen software (2008–2010) (Carl Zeiss).

### Immunoprecipitations

Embryos injected with mRNA coding for Wnt8a–HA and LRP6–Myc, Wnt11b–HA and Ror2–Myc, or Wnt3a–HA and LRP6–Myc were homogenised in RIPA buffer [137 mM NaCl, 20 mM Tris-HCl pH 8, 2 mM EDTA, 1% (v/v) NP40 replacement, 1% protease inhibitors, 1% phosphatase inhibitors]. Clarified extract was incubated with either 1∶1000 rabbit anti-HA antibody (Abcam, AB9110) or 1∶250 rabbit anti-Myc (Abcam, AB9106) antibody, in the presence of 1% BSA (Sigma, A3059) overnight at 4°C followed by a 2-hour incubation with protein-A–Sepharose beads (Peirce, 20333). After extensive washing, samples were eluted by boiling in Llamelli buffer prior to SDS-PAGE.

### Western blotting

For western blotting, animal caps were homogenised in (20 mM Tris-HCl pH 7.5, 50 mM NaCl, 1 mM EGTA, 2 mM MgCl_2_) using an end-over-end invertor (Rotamix RM1) at 4°C for 10 min and centrifuged at 1000 ***g*** for 10 min at 4°C. The supernatant was boiled in Llamelli buffer prior to SDS-PAGE and transfer onto a PDVF membrane.

Western blots were performed according to standard protocols. Primary antibodies used were mouse anti-HA.11 (Clone 16B12, Covance MMS-101P), rabbit anti-Myc (Abcam, AB9106), and mouse anti-β-catenin antibodies. Secondary antibodies used were sheep anti-mouse-IgG antibody conjugated to horseradish preoxidase (HRP; Amersham, NA931) and mouse anti-rabbit-light-chain antibody conjugated to HRP (Jackson, 211-032-171).

### Statistical analysis

Discrete data were analysed using the Chi squared test, continuous data was analysed using Mann–Whitney U test (*P<0.05, **P<0.01). The percentage colocalisation of different fluorescent proteins with the plasma membrane was determined using a program written in Matlab. In brief, the program calculates the percentage of green pixels colocalising with mRFP pixels, removes these pixels and then calculates the percentage of black pixels (cytoplasmic) colocalising with green pixels. This allows the relative amount of protein colocalising with the cell membrane to be determined. The range of Wnt8a– and Wnt11b–GFP diffusion was analysed using the ROI manager function of FIJI Image J. This allowed the average pixel intensity of fluorescent Wnt ligands to be plotted. Curves were fitted to the data using the regression wizard in Sigmaplot 12.5. All of the plots were fitted using a single exponential decay model with three parameters. The curves where then used to determine the rates of ligand decay with increasing distance from the source.

## Supplementary Material

Supplementary Material
