# Supplementary material for: Sulf1 has ligand-dependent effects on canonical and non-canonical Wnt signalling
Source: J Cell Sci. 2015 Apr 1;128(7):1408–21. doi: 10.1242/jcs.164467 (PMC4379729; doi:10.1242/jcs.164467)
Supplement: Supplementary Material [file supp_128_7_1408__index.html]

Sulf1 has ligand-dependent effects on canonical and non-canonical Wnt signalling — Supplementary Material 

# Sulf1 has ligand-dependent effects on canonical and non-canonical Wnt signalling

## JCS164467 Supplementary Material

**Files in this Data Supplement:**

- **Supplementary Material**
