# Supplementary material for: Sulf1 has ligand-dependent effects on canonical and non-canonical Wnt signalling
Source: J Cell Sci. 2015 Apr 1;128(7):1408–21. doi: 10.1242/jcs.164467 (PMC4379729; doi:10.1242/jcs.164467)
Supplement: Supplementary Material [file supp_128.7.1408_JCS164467.pdf]

Supplemental Data

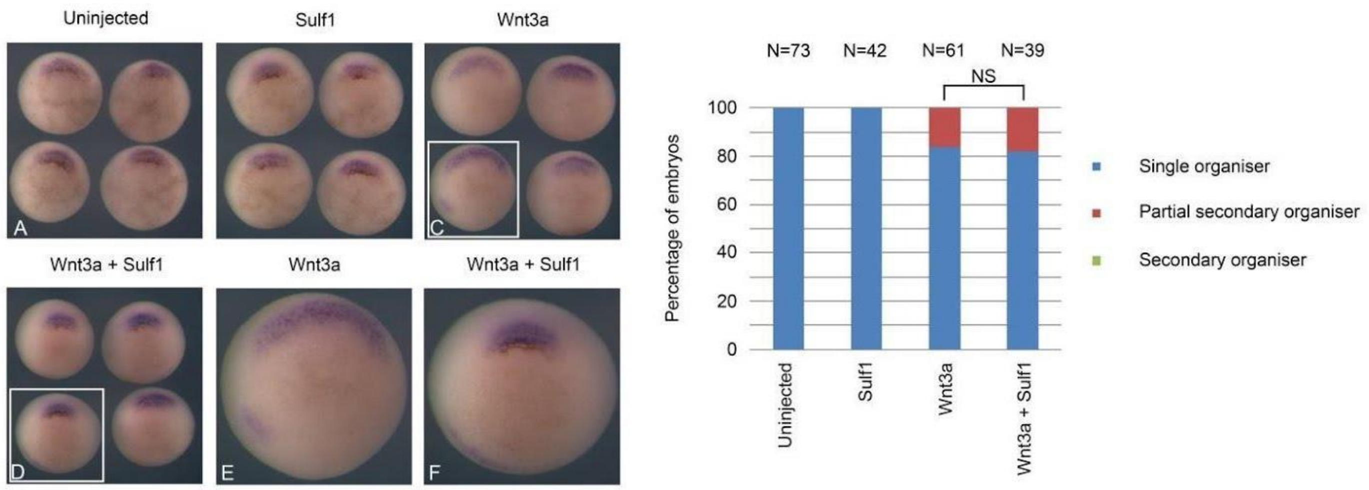

Figure 1

[A-F] *Xenopus laevis* embryos were microinjected with mRNA encoding *Wnt3a* (0.1pg) and *Sulf1* (1ng) into a single ventral blastomere at the four cell stage. *In situ* hybridisation for the gene *chordin* was performed at NF stage 10.5. [A] Uninjected control embryos. [B-D] Embryos injected with [B] *Sulf1*, [C] *Wnt3a* or [D] *Sulf1* and *Wnt3a*. The white boxes in [C] and [D] were used to create panels [E] and [F] respectively. [G] The data shown in [A-F] is quantified in [G], Chi squared test (NS=not significant), N=number of embryos.

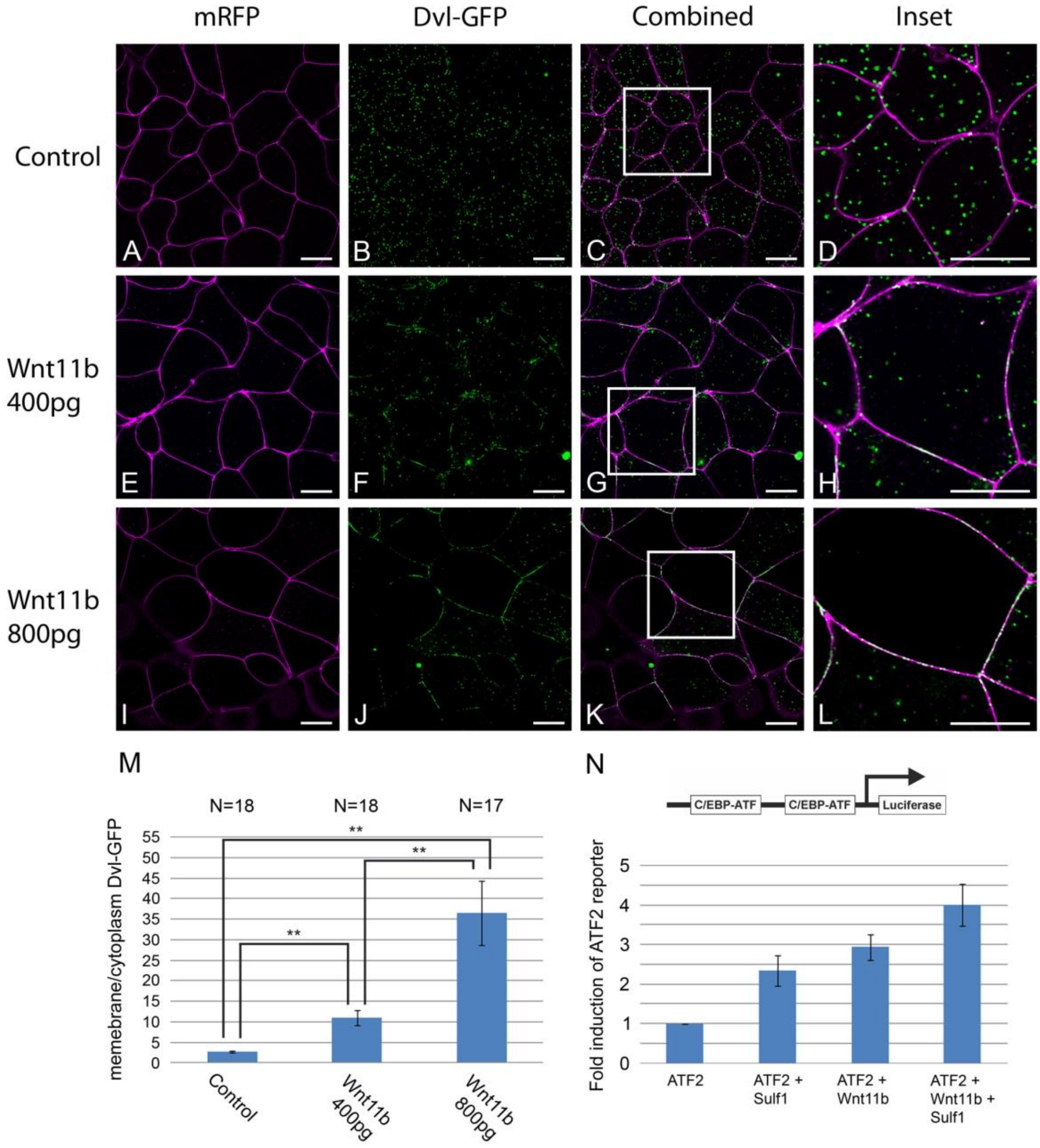

Figure 2

[A-L] *Xenopus laevis* embryos were microinjected bilaterally with mRNA encoding *mRFP* (500pg) and *Dvl-GFP* (500pg) into the animal hemisphere at the two cell stage. In addition embryos were injected with increasing amounts of *Wnt11b* mRNA. [A-D] Control animal explants over expressing mRFP and Dvl-GFP. Animal explants injected with [E-H] *Wnt11b* (400pg) or [I-L] *Wnt11b* (800pg) mRNA. The white boxes in [C], [G] and [K] mark the areas used to create panels [D], [H] and [L] respectively. [M] The data shown in [A-L] is quantified in [M]. Quantification was done using a program written in MatLab, Mann-Whitney U (**P<0.01), error bars represent s.e.m, N=number of embryos. mRFP (magenta), Dvl-GFP (green), scale bars represent 20um. [N] A diagram of the ATF2 reporter used for this assay is shown above a graph depicting the results obtained when 100pg of ATF reporter plasmid DNA and 1pg of Renilla was injected into the marginal zone of all four cells of four cell stage *Xenopus laevis* embryos. In addition embryos were injected with mRNA encoding Sulf1 (4ng) or Wnt11b (200pg) with a control mRNA or with Wnt11b (200pg) together with Sulf1. Embryos were lysed and analysed in a luminometer at stage 10 to assay luciferase activity. The graph illustrates the effects of Sulf1 and Wnt11b on the activation of the ATF reporter.

Supplemental Data

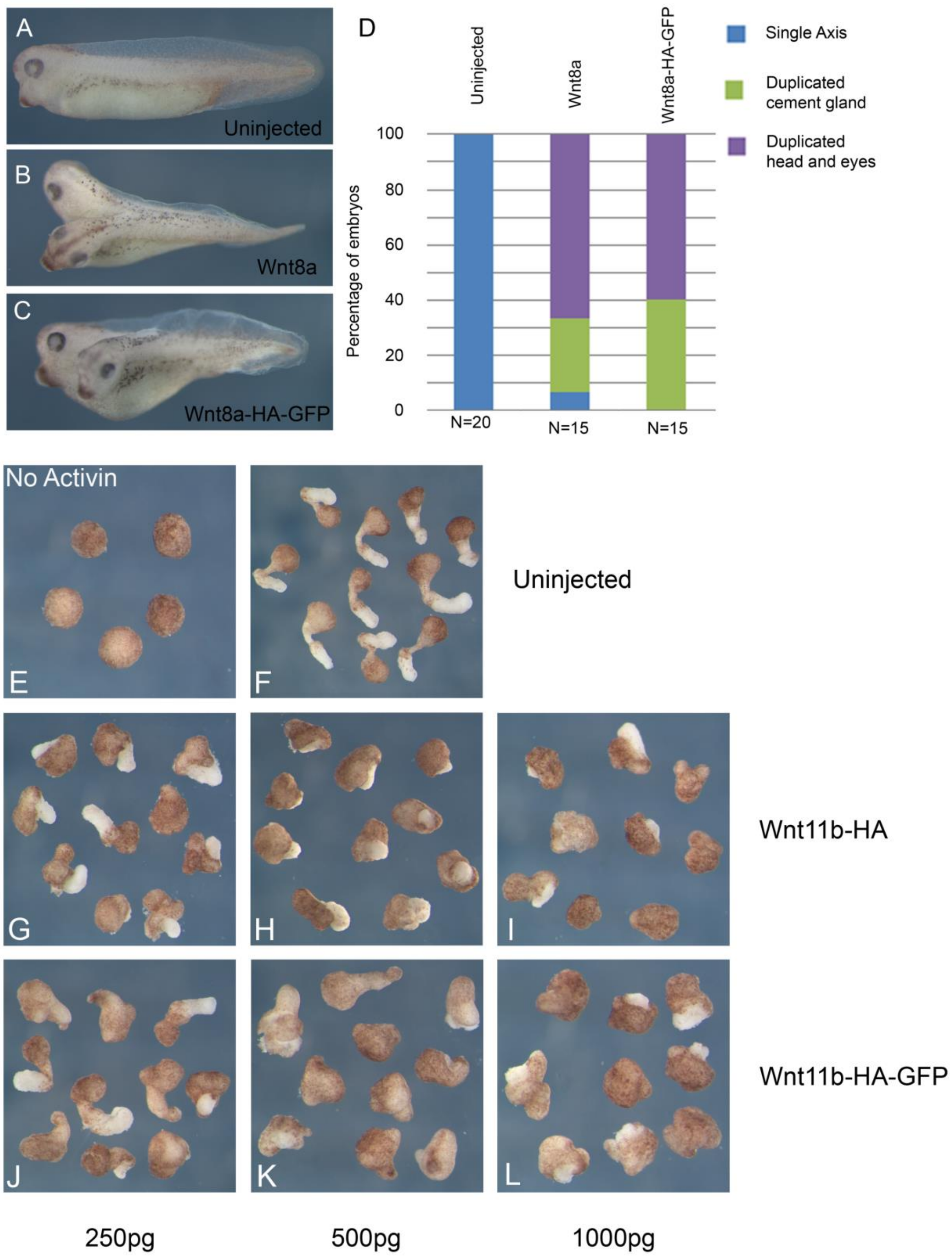

Figure 3

Supplemental Data

Figure 3

Wnt8a-GFP and Wnt11b-GFP are biologically active.

mRNA encoding *Wnt8a* (10pg) or *Wnt8a-HA-GFP* (10pg) was injected into one ventral blastomere of an embryo at the four cell stage. Embryos were cultured until NF stage 38 and then examined for phenotype. [A] Lateral view of an uninjected embryo. [B]. An example of an embryo injected with Wnt8a. [C]. An example of an embryo injected with Wnt8a-HA-GFP.[D]. Graph depicting the quantification of the frequency of axis duplication in embryos over-expressing Wnt8a and Wnt8-HA-GFP. Wnt8a-HA-GFP showed a similar level of activity to Wnt8a in axis duplication assays.

mRNA encoding *Wnt11-HA* or *Wnt11-HA-GFP* was injected bilaterally into the animal hemisphere of embryos at the two cell stage. Embryos were cultured until NF stage 8 and then animal cap explants were explanted and cultured in either the presence or absence of activin until NF stage 19. [A-B] Uninjected animal caps cultured in either the absence [E] or presence [F] of activin. [G-I] Animal caps treated with activin and injected with increasing amounts of *Wnt11b-HA*. [H-L] Animal caps treated with activin and injected with increasing amounts of *Wnt11b-HA-GFP.* Over-expression of increasing amounts of Wnt11-HA or Wnt11-HA-GFP inhibited activin induced convergent extension of animal caps to a similar extent.

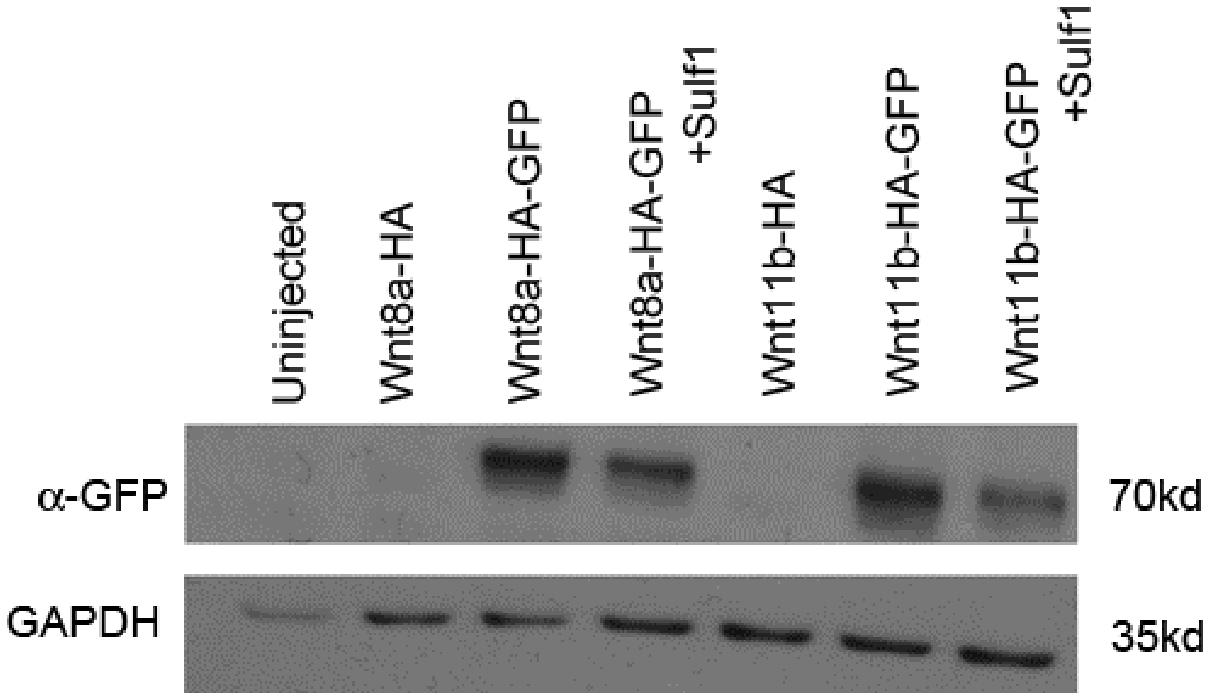

Figure 4

Expression of Wnt8a-GFP and Wnt11b-GFP in Xenopus animal caps.
Western blot analysis of *Xenopus* embryos injected with 400ng of mRNA coding for Wnt fusion proteins (Wnt8a-HA-GFP or Wnt11b-HA-GFP) together with 4ng of mRNA coding for Sulf1 or the control LacZ. Wnt proteins are detected at similar levels in the presence and absence of Sulf1.
